# First Reported Case of Iatrogenic Cardiac Tamponade Following Chest Drain Insertion for Tension Pneumothorax in a Premature Newborn

**DOI:** 10.1155/crpe/7960753

**Published:** 2025-05-18

**Authors:** Daniel Grandmougin, Pan Dan, François Wurtz, Olivier Larmure, Constanta Birliga, Jean-Marc Jellimann, Nathan Giroux, Gilles Bosser, Juan-Pablo Maureira

**Affiliations:** ^1^Department of Cardiac Surgery and Transplantation, ILCV Louis Mathieu, Centre Hospitalier Régional et Universitaire de Nancy, Nancy, France; ^2^Department of Anesthesia, Hôpital d'Enfants, Centre Hospitalier Régional et Universitaire de Nancy, Nancy, France; ^3^Department of Pediatric Surgery, Hôpital d'Enfants, Centre Hospitalier Régional et Universitaire de Nancy, Nancy, France; ^4^Department of Neonatology, Hôpital d'Enfants, Centre Hospitalier Régional et Universitaire de Nancy, Nancy, France; ^5^Department of Congenital Heart Diseases and Pediatric Cardiology, Hôpital d'Enfants, Centre Hospitalier Régional et Universitaire de Nancy, Nancy, France

**Keywords:** cardiac tamponade, chest drain, myocardial penetrating injury, pericardial effusion, pneumothorax, premature newborn

## Abstract

Cardiac tamponade is a challenging clinical situation in preterm newborns. We report the first case of an iatrogenic cardiac tamponade secondary to direct myocardial disruption with pericardial penetration following unsuccessful attempts to drain a right tension pneumothorax in a 34-week premature female newborn. The pathophysiologic mechanisms involved are discussed.

## 1. Introduction

Prematurity remains the most common cause of neonatal mortality. Preterm newborns are at greater risk of morbimortality with specific complications such as respiratory distress syndrome (RDS), brain injury, intraventricular hemorrhage, necrotizing enterocolitis [1], and iatrogenic complications such as pericardial effusion resulting from indwelling venous catheters used in neonatal cares [2]. This latter complication may lead to life-threatening situations requiring emergency treatment with either echo-guided pericardiocentesis or surgical drainage. Pneumothorax occurs more frequently in the neonatal period than at any other time of life [3]. Its management includes either a conservative approach with oxygen supplementation and administration of pulmonary surfactant, or more invasive procedures such as needle exsufflation and chest tube drainage [3]. Complications arising from pneumothorax drainage are multiple with subcutaneous emphysema, infection, tube misplacement, lung perforation [4], bleeding (intercostal vessels and mammary artery), atelectasis, drain occlusion, and pain [5]. In adults, severe complications have been rarely reported with perforation of the left ventricle [6], the liver and inferior vena cava [7], the pulmonary artery [8], and misplacement of the drain into the hepatic vein [9].

Herein, we report a rare case of cardiac tamponade secondary to myocardial disruption after insertion of a chest drain in a preterm female baby who was successfully managed with urgent surgical pericardial drainage. The relevant medical literature is reviewed.

## 2. Case Presentation

A 2650-g premature newborn female was delivered at 34 weeks gestation via cesarean section due to preeclampsia. Birth length was 48 cm and head circumference 33 cm. Blood biochemistry, cell counts, and coagulation screenings were within the normal ranges. Four hours after birth, administration of surfactant therapy was implemented for a membrane hyaline disease with a noninvasive respiratory support including titrated oxygen therapy.

Unfortunately, one day after birth, she needed to be transferred to our university maternity hospital to treat a severe RDS caused by a right tension pneumothorax for which several attempts to drain the pneumothorax with needle thoracocentesis had failed. Therefore, prior to the transfer, the baby required endotracheal intubation with placement of a chest tube through the 5^th^ intercostal space in the right anterior axillary line and an umbilical venous catheter (UVC).

**Figure 1:**
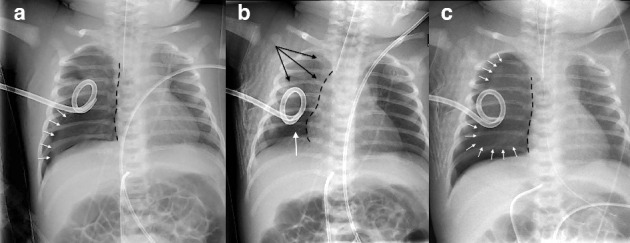
(a) Chest radiography performed after admission to the University Maternity Hospital, showing the first thoracic drain inserted 30 min after birth, with a right recidive pneumothorax (white arrows). The right atrial silhouette is translated to the left by the right collapsed lung (black dotted line), illustrating cardiac compression. (b) Residual right base-thoracic pneumothorax after mobilization of the drain and evacuation of 160 mL of air. Black arrows show expansion of the upper and median parts of the lung. The right atrial silhouette is no more left translated (black dotted line). (c) Complete right recidive pneumothorax (white arrows) with a recurrence of the left translation of the right atrial silhouette (black dotted line).

Immediately after admission, recurrence of RDS was observed with desaturation necessitating important oxygen requirements. Chest radiography confirmed a complete right recidive pneumothorax despite the chest drain (Figure 1(a)). The drain was then mobilized and 160 mL of air was evacuated. Nevertheless, chest roentgenogram showed a residual right basi-thoracic pneumothorax (Figure 1(b)). Few hours later, RDS worsened, displaying a no bubbling drain and an increasing pneumothorax (Figure 1(c)). The drain was first mobilized with no radiological and clinical improvement. Therefore, decision was made to insert a new drain through the 5^th^ intercostal space in the right midaxillary line after removal of the previous one inserted in the 5^th^ intercostal space in the right anterior axillary line. The first attempt was unsuccessful and at the second attempt, while the mandrel of the drain was pulled out after drain insertion, an important bleeding into the drain was observed. The drain was immediately removed while a rapid hemodynamic deterioration occurred with cutaneous pallor, hypotension (mean arterial pressure (MAP): 22 mm Hg), and bradycardia (heart rate: 80–100 bpm), necessitating a bolus of atropine (10 μg/kg).

Regarding etiology of the acute hemodynamic decompensation, a transthoracic echography revealed a cardiac tamponade with alteration of contractility, hypovolemia, and a right pleural effusion leading to suspect a myocardial injury. Then, units of O negative red blood cells were transfused to the neonate (20 mL/kg) in order to treat hypovolemia and correct anemia as well as fresh frozen plasma (10 mL/kg) and vitamin K (5 mg) (prothrombin: 40%; normal level > 70%).

Dobutamine infusion (10 μg/kg/min) was started with the administration of 2 normal saline boluses (10 mL/kg), resulting in a moderate and transient hemodynamic improvement (MAP: 37–45 mm Hg/heart rate: 110–123 bpm). Therefore, after discussion with the surgical team, in order to avoid delay of the surgical management, the CT scan, previously scheduled, was canceled and the baby was rapidly transferred to the department of pediatric surgery for pericardial and thoracic drainages.

**Figure 2:**
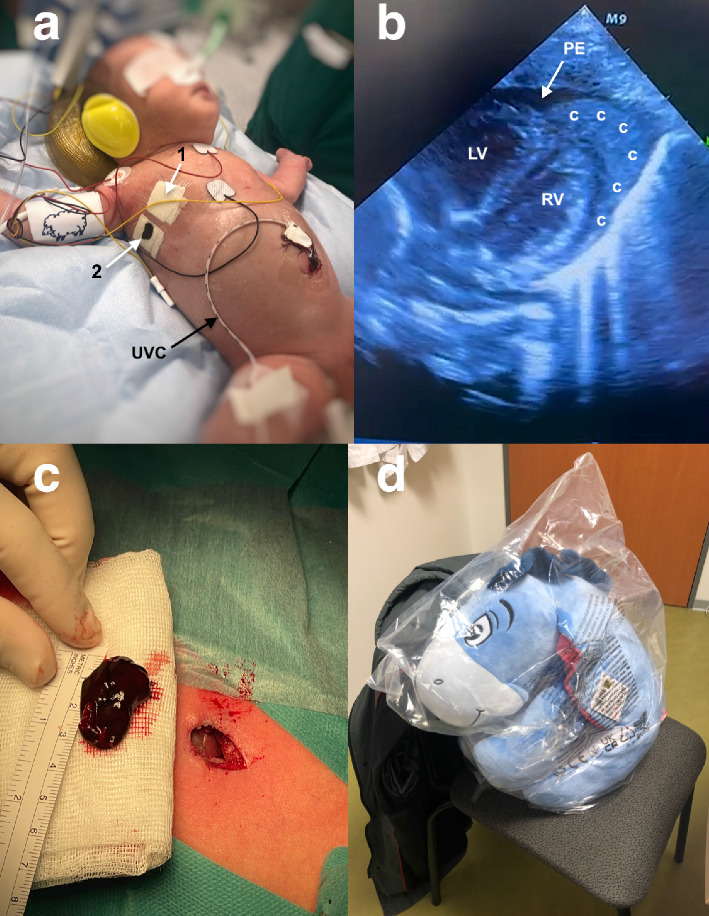
(a) View of the intubated baby prior to surgery. Arrow 1 shows the gauze covering the site of insertion of the first drain. Arrow 2 shows the gauze covering the bleeding tube thoracostomy after removal of the second drain (UVC: umbilical venous catheter). (b) Cardiac echography performed in the operative room, prior to surgery, displays a pericardial effusion (PE), with a huge intrapericardial compressive clot (c) over the cardiac apex and extending along the right ventricle (RV) (LV: left ventricle). (c) Operative view of the 3.3 cm clot after removal with the subxiphoid skin incision. (d) New comforting friend named Eeyore discharged with the cute baby after surgery.

During the transfer, hemodynamic instability rapidly worsened with low arterial pressure (MAP: 23 mmHg), requiring an increased dose of dobutamine to 15 μg/kg/min and introduction of noradrenaline (0.2 μg/kg/min). After installation and monitoring of the newborn on the operative table (Figure 2(a)), a repeat cardiac echography confirmed a newly appeared right hemothorax with a worsening cardiac tamponade and presence of a compressive clot along the right ventricle (Figure 2(b)/Video 1). We then decided to perform emergency surgical pericardial drainage with the removal of the clot and pleural drainage.

Surgery was performed through a subxiphoid access. After a rapid exposure of a distended pericardium, a pericardiostomy evacuated blood resulting in a moderate improvement of hemodynamics whilst the compressive clot was still into the pericardial cavity. A 3.3 cm clot was then visualized along the right ventricle and gently removed (Figure 2(c)/Video 2), allowing to restore hemodynamic stability and rapidly wean the baby from inotropic support. The right pleural hemopneumothorax was drained through the same access. After a complete cardiac decompression, we carefully checked that no bleeding was still active from the right ventricle and two 10 Fr. drains were inserted into the pleural and pericardial cavities, respectively. We then delayed surgical closure for 20 min to secure hemodynamic stability. Postoperative course was uneventful and repeat echocardiograms showed no residual pericardial effusion. Both drains were removed on the 3^rd^ postoperative day. The baby was discharged to her birth maternity hospital on the 10th postoperative day with her new comforting stuffed friend Eeyore (Figure 2(d)) with neither recurrence of pneumothorax nor residual pericardial effusion.

## 3. Discussion

Over the last decades, therapeutic progresses in neonatology improved the overall survival of premature newborns. Among these progresses, maintaining long-term indwelling central venous catheters (CVCs) used to optimize volemia and administer both pharmacologic drugs and parenteral nutrition in order to sustain growth remains a challenge necessitating specific skills. If the placement of CVC is a routine maneuver in neonatal intensive care units (NICU), nevertheless it is not risk-free as several complications have been reported among which is cardiac tamponade [10–13]. This latter complication is rare with an incidence ranging from 0.07% to 2% [11, 12], nevertheless with a mortality rate as high as 50% [2, 12–15]. Among venous catheters used in NICU, UVCs are routinely and safely utilized [16–18] in preterm infants with obvious and specific advantages [19]. However, it is interesting to observe that the first case of CVC-related pericardial effusion published in 1972 by Daly Walker involved UVC [20].

In preterm infants, adverse events may occur among which pericardial effusion, cardiac tamponade, and pneumothorax are life-threatening situations prompting early diagnosis and urgent management.

Owing to specific anatomic and cellular characteristics supporting myocardial and pericardial fragility, preterm newborns are at a greater risk of myocardial damage and pericardial effusion [14, 21].

Main etiologies of pericardial effusions, reported in neonatology, are iatrogenic and mostly result from indwelling CVC [10–14, 22–24]. Thus, myocardial damages may result from catheter misplacement into cardiac cavities with myocardial disruption and pericardial effusion. However, Warren et al. [14] described a series of 5 autopsies performed in cases with sudden death from pericardial effusion in neonates with CVCs and appropriate CVC positioning. Then, they hypothesized that the hyperosmolarity of parenteral nutrition might have damaged myocardial wall leading to pericardial effusion.

Pericardial effusion may be asymptomatic or it may lead to hemodynamic instability when the cardiac function is compromised and hence by cardiac tamponade. This condition can be fatal if decompression is not done timely, either with ultrasound-guided pericardiocentesis or surgical pericardiostomy.

Theoretically, cardiac tamponade and chest drainage have no causal relationship as confirmed in a comprehensive literature search, completed in PuBMed database using the terms  « pericardial effusion after/following drainage of pneumothorax/chest drainage, cardiac tamponade after/following drainage of pneumothorax/chest drainage » where no report of cardiac tamponade due to direct myocardial injury following drainage of pneumothorax in preterm neonate, was found throughout the last 3 decades. Only one case, published in 1993 [25], mentioned a pericardial effusion in a premature baby due to indirect myocardial injury with no penetration of the pericardium caused by chest tube insertion.

Hence, our case is the first report of cardiac tamponade due to direct myocardial disruption caused by the mandrel of a chest drain following attempts to drain pneumothorax in a premature infant.

Drainage of neonatal pneumothorax can be achieved by needle aspiration (thoracentesis) or by chest tube thoracostomy [26, 27].

Thoracentesis consists of aspiration of air with a 10 mL–20 mL syringe through a needle or an angiocatheter. The needle is inserted into the second or third intercostal space in the midclavicular line. Flow of air into the syringe confirms that the pneumothorax has been reached by the needle, which should not be inserted further to avoid lung damage. Tube thoracostomy is performed by insertion of a chest drain (10 or 12 Fr.), usually in the 4th or 5th intercostal space in the midaxillary line. A mandrel is usually used to facilitate the penetration of the drain. If this approach is known to be associated with risk of lung injury [4], conversely, the risk of direct myocardial injury with penetration of the pericardium is not at all described in the literature as mentioned above. In preterm newborn, since the distance between the cutaneous site of drain insertion and pericardium is short, both drain path and depth need to be carefully planned and adapted in order to avoid pericardial perforation and myocardial injury, highlighting the valuable interest of ultrasonic guidance to reduce the complication rate during drain placement.

Retrospectively, in our case, we assumed two hypotheses linking pneumothorax drainage and acute cardiac tamponade.

1. The drain was not advanced through the pericardium. Therefore, tamponade might have resulted from damage of epicardial vessels of the heart caused by direct external contact of the drain with the pericardium. However, the newly diagnosed hemothorax and bleeding into the drain do not support this hypothesis since there was no communication between the pericardial cavity and the drain and hence no possibility for the pericardial blood to be evacuated through the lumen of the chest drain.
2. The drain with the mandrel was advanced through the thin pericardium and directly damaged the myocardium with a subsequent bleeding into the pericardial cavity. Thereafter, rapid administration of both fresh frozen plasma and vitamin K likely contributed to reduce bleeding and in part promote clotting into the pericardium. This hypothesis is the most consistent, supported by the sudden and important bleeding into the drain after removal of the mandrel, the newly detected hemothorax, the rapid hemodynamic decompensation, and the importance of both hypovolemia and pericardial effusion.

The less invasive surgical access through the subxyphoid approach for the decompression of the cardiac tamponade in this case was chosen because of the hemodynamic instability, the formation of the pericardial effusion with a compressive clot, and a concomitant hemopneumothorax. The other option with right thoracotomy involving rib spreading was considered too invasive with more adverse consequences on the pulmonary function. In addition, if a wider exposure to gain enhanced visibility is necessary, the subxyphoid approach easily allows extension to a lower hemisternotomy.

This case reminds that the insertion of a chest drain in premature newborn requires a careful awareness, as it is not a harmless and risk-free procedure due to specific anatomic characteristics. Therefore, while insertion of a chest drain has been formally decided, ultrasound guidance to optimize drain placement as well as a systematic cardiac echography to monitor a potential subsequent pericardial effusion following chest drain insertion should be systematically implemented to prevent a cascade of pejorative events.

## 4. Conclusions

Cardiac tamponade remains a rare but serious complication of pneumothorax drainage in newborn infants and an accurate knowledge and awareness of clinicians regarding this complication is crucial to organize a reactive and optimal therapeutic management. Since specific anatomic characteristics support risks of cardiac damage in premature newborns, image-guided drainage of the pleural space for accurate placement of a drain or an angiocatheter should always be considered [28, 29] and may contribute to reduce incidence of complications [30].

## Data Availability

The data used to support the findings of this study are available from the corresponding author upon request.

## Nomenclature

RDS: Respiratory distress syndrome
UVC: Umbilical venous catheter
MAP: Mean arterial pressure
BPM: Beats per minute
CVC: Central venous catheter
NICU: Neonatal intensive care unit

## Supporting Information

Supporting Information 1 The video 1 shows the preoperative cardiac echography displaying the pericardial effusion with a compressive clot trapped between the pericardium and the right ventricle. Video 1: https://drive.google.com/file/d/1xEvIq7cIzioJRbqWz8NSX9382YSLzdNZ/view?usp=share_link.

Supporting Information 2 The video 2 shows the evacuation of the compressive clot from the pericardial cavity through a subxiphoid pericardiostomy. Video 2: https://drive.google.com/file/d/1zdaY1D8NdJJr6j9k9b3agH9mtsPvlebF/view?usp=share_link.
